# Peer influence: neural mechanisms underlying in-group conformity

**DOI:** 10.3389/fnhum.2013.00050

**Published:** 2013-03-08

**Authors:** Mirre Stallen, Ale Smidts, Alan G. Sanfey

**Affiliations:** ^1^Department of Marketing Management, Rotterdam School of Management, Erasmus UniversityRotterdam, Netherlands; ^2^Centre for Cognitive Neuroimaging, Donders Institute for Brain, Cognition and Behaviour, Radboud UniversityNijmegen, Netherlands

**Keywords:** conformity, in-group bias, MRI imaging, judgment and decision-making, social influence

## Abstract

People often conform to the behavior of others with whom they identify. However, it is unclear what fundamental mechanisms underlie this type of conformity. Here, we investigate the processes mediating in-group conformity by using functional magnetic resonance imaging (fMRI). Participants completed a perceptual decision-making task while undergoing fMRI, during which they were exposed to the judgments of both in-group and out-group members. Our data suggest that conformity to the in-group is mediated by both positive affect as well as the cognitive capacity of perspective taking. Examining the processes that drive in-group conformity by utilizing a basic decision-making paradigm combined with neuroimaging methods provides important insights into the potential mechanisms of conformity. These results may provide an integral step in developing more effective campaigns using group conformity as a tool for behavioral change.

## Introduction

People are often influenced by others with whom they identify. They buy clothes similar to those of their peers, visit restaurants because their colleagues go there, and download music their friends listen to. By adopting the tastes of others, people show they belong to a specific group. This social factor, whereby people follow the behavior or advice of others they associate with, has been labeled *in-group influence*. It is not limited to product choice (Bearden and Etzel, 1982; Berger and Heath, 2007, 2008), but influences behavior even when identity signaling is not an issue. For instance, a field experiment on household energy conservation showed that informing people about their neighborhood's average home energy usage resulted in a change in household energy consumption, specifically toward the mean of their neighborhood (Schultz et al., 2007). Similarly, a study on conservation behavior found that hotel guests were more likely to reuse towels when informed that guests who had stayed in that same room had reused towels than if they were informed about the behavior of guests in general (Goldstein et al., 2008).

Given the powerful influence of the in-group, it therefore comes as no surprise that there has been an increase in the use of group conformity as a tool for behavioral change. People often overestimate both the degree of approval and the prevalence of negative behavior among peers, such as drinking, drug use, violence, littering, or cigarette smoking (Baer et al., 1991; Donaldson et al., 1994; Schultz, 1999; Neighbors et al., 2004; Berkowitz, 2010). Social influence-programs seek to correct these misperceptions by exposing their target groups to the actual attitudes of their peers and the real frequency of the undesirable behaviors. However, despite the initial popularity of these programs, the evidence for their success in establishing behavioral change has been mixed. Over time, many programs failed to change behavior substantially (Peeler et al., 2000; Clapp et al., 2003), and some social influence-programs even showed effects of increasing the undesirable behavior they tried to reduce (Granfield, 2005; Wechsler et al., 2003). The mixed findings on the effectiveness of social influence-programs demonstrate that it is still unclear exactly what psychological processes may mediate in-group conformity. In order to understand why and when people conform to their in-group, we need to understand the mechanisms that drive in-group conformity.

The aim of the present study was to gain greater insight into the processes underlying in-group conformity. To examine the mechanism of in-group conformity, we used functional magnetic resonance imaging (fMRI), a modern neuroscientific method that provides a non-invasive measure of neural activity by assessing regional changes in blood oxygenation [blood oxygen level dependent (BOLD) response]. Using fMRI enables us to make inferences about the processes that underlie in-group conformity, which is difficult to assess using behavioral measures alone. In addition, to investigate the basic underlying processes, we measured in-group conformity using an artificial group manipulation and using a domain that was neither relevant for identity signaling nor related to actual choice. Examining the neural processes driving in-group conformity under these minimal conditions provides fundamental insights into the basic brain mechanisms, and may help in designing more effective social norm campaigns.

Although the application of neuroimaging methods in decision-making research has increased in popularity during the last decade, only recently have neuroscientists started to identify the brain networks implicated in social influence, for example examining the influence of experts (Klucharev et al., 2008; Campbell-Meiklejohn et al., 2010), the persuasiveness of celebrities (Stallen et al., 2010), the mechanisms of racial bias (Beer et al., 2008; Van Bavel et al., 2008; Gonsalkorale et al., 2011), the influence of majority behavior on individual decision-making (Berns et al., 2005, 2010; Klucharev et al., 2009, 2011; Mason et al., 2009), and, most relevant to the current investigation, the influence of in-group membership on both money allocation (Volz et al., 2009) and helping behavior (Hein et al., 2010). Volz and colleagues (2009) investigated the neural implementation of social identity theory, which assumes that each individual has both a personal and a social identity, and that the way information is processed depends on which identity of the individual is salient at the time of decision-making (Tajfel and Turner, 1986). The results of Volz and colleagues (2009) support social identity theory by demonstrating that the social self is derived from the same cognitive processes as the individual self, as activation of both types of identities resulted in similar neural patterns in the prefrontal and parietal network. A second study on in-group influence by Hein and colleagues (2010) investigated the neurobiological basis of the decision to help either an in-group or out-group member in pain. Their results showed that seeing an in-group member in pain evoked more empathy-related responses in the brain than seeing an out-group member in pain, and demonstrated that the degree of this empathy-related response predicted in-group favoritism in actual helping behavior at a later point in time. Importantly however, none of these studies on social influence in the brain examined the processes that underlie conformity to the in-group.

## Materials and methods

### Participants and design

Twenty-eight healthy right-handed participants (mean age 20.7 years) took part in the experiment. All were free of neurological or psychiatric illness, head trauma or drug abuse, and none were taking medication. Written informed consent was obtained according to the local medical ethics committee, and participants were compensated financially. Data from three participants were discarded due to technical problems, and one participant was excluded because he guessed the study aim. This resulted in 24 subjects for final analyses (12 males). We used a repeated measures design with the identity of the group member (in-group or out-group member) as a single within-subject factor.

### Materials and methods

Subjects arrived alone to the experiment. Upon arrival, participants' group membership was manipulated using a minimal group paradigm approach (Tajfel et al., 1971). In the task, adapted from Volz et al. (2009), five perceptual illusions, such as the young girl-old woman illusion, were shown for 2 s each, After each illusion, two possible answers were displayed on the screen and participants were asked to choose between them. Then, participants were informed that they had been categorized as people who either focus on the foreground of visual illusions, people who focus on the background, or as people who could not be classified into either of these two categories. Unbeknownst to participants, everyone was classified as a foreground perceiver (in-group). The other two groups (background and unclassified) will be referred to as the out-group. We manipulated group membership artificially instead of using real, existing groups, as this allowed us to control the (minimal) information participants had about their in-group and out-group members, and hence ensured that the hypothesized in-group conformity effect could not be explained by factors other than group membership, such as for example perceived differences in expertise in perceptual decision-making.

### Decision-making task

**Figure 1:**
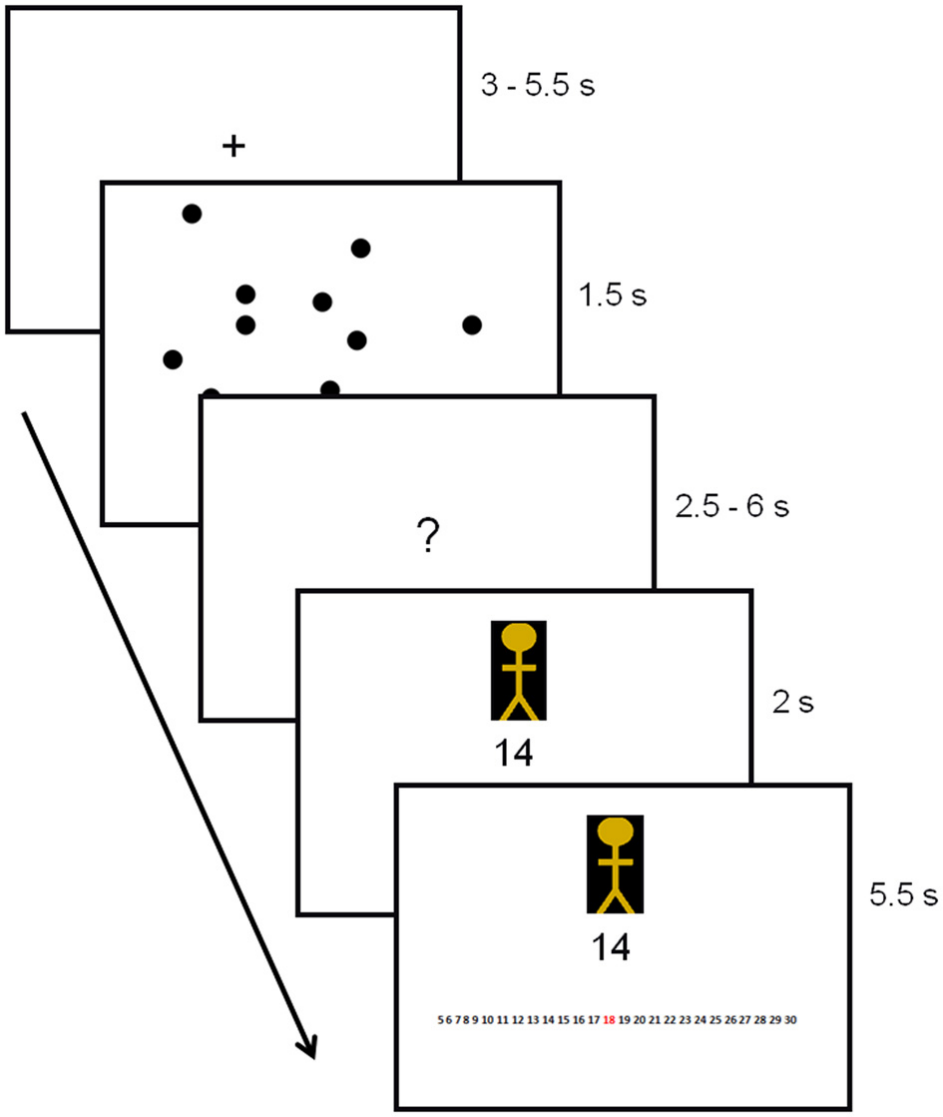
**Trial outline with duration times in seconds.** Group membership of the other participant was indicated by the colored cartoon.

After the perceptual illusion task, participants completed the decision-making task while undergoing MRI (Figure 1). First, participants were instructed to look at a dot pattern on a computer screen for 1.5 s. The number of dots on display ranged from 5 to 30 (*M* = 15, *SD* = 7.5), and the participants' goal was to estimate the number of dots as accurately as possible. The number of dots used was based on pre-tests conducted with a different set of participants (*N* = 42). Pre-tests showed that, on average, participants were able to estimate about 11 dots (*SD* = 2.2 dots) correctly within 1.5 s. Because we required our experiment to include both easy and difficult trials (easy trials were included to ensure motivation), we varied the number of dots from 5 to 30 across trials. After the brief presentation of the dots, participants were instructed to think about their estimate (duration jittered between 2.5 and 6 s). Next, the estimate of a previous participant was displayed. This estimate came from either a member of the same group as the participant, that is, a foreground perceiver (in-group member) or from a member of a different group (out-group member). Group membership of the other participant (foreground perceiver, background perceiver or unclassified perceiver) was indicated by a colored cartoon of either yellow, purple or blue. Colors were counterbalanced to ensure no confound between the color of the cartoon and group membership.

After presentation of the estimates, a response screen appeared. This screen was identical to the previous screen on which the estimate was presented, except for a response bar displayed at the bottom of the screen. This bar consisted of a row of numbers from 5 to 30, on which participants were instructed to indicate their estimate. Responses were indicated by scrolling to the number of their choice and pressing a confirmation button. The estimates provided by in-group and out-group members were predetermined by a computer script and, unbeknownst to the participant, were always correct. Finally, to enhance motivation, participants were told that the group who performed best would win an (unspecified) prize, with the winning group notified at the end of the study.

The presentation of a fixation screen (duration jittered between 3.5 to 5 s) signaled the start of a new trial. Participants performed 214 trials. To maintain attention, 6 self-paced breaks were included. The total scanning session took approx. 55 min.

### MRI acquisition parameters

Functional images were acquired with a 1.5T Siemens Sonata scanner, using an ascending slice acquisition and a T2^*^-weighted echo-planar imaging (EPI) sequence (TR 2.29 s, TE 30 ms, flip angle 70°, slice matrix 64 × 64 mm, slice thickness 3.0 mm, slice gap 0.5 mm, FOV 224 mm). Anatomical scans were acquired with a T1-weighted MP-RAGE sequence (176 sagittal slices, TR 2.25 s, TE 3.93 ms, flip angle 15°, slice matrix 256 × 256, slice thickness 1.0 mm, no gap, FOV 256 mm).

### Dependent variables

#### Behavioral questionnaires

To test the group manipulation, participants answered a questionnaire at the end of the experiment. This measured the level of identification (“I feel connected to the blue/yellow/purple team”), trust (“I trust people from the blue/yellow/purple team”), and the degree of positive associations (“I have positive associations with the blue/yellow/purple team”) toward other participants. Responses ranged from 1 (not true at all) to 7 (very true).

#### Conformity

Conformity was assessed by calculating the percentage of trials on which participants gave the same judgments as the in-group or out-group member.

#### Brain imaging analyses

Data were preprocessed and analyzed using a standard software package (SPM8, Wellcome Department of Cognitive Neurology London). The first 5 images of each participant's EPI sequence were discarded to allow for longitudinal relaxation time. The remaining images were realigned to the first imaging volume. Functional images were corrected for motion and differences in slice time acquisition. Next, images were normalized to the Montreal Neurological Institute (MNI) template using parameters defined from the normalization of the anatomical scan to the MNI template, and images were smoothed with a Gaussian kernel of 8 mm full-width at half-maximum to reduce noise. Motion parameters were stored and used as nuisance variables in the general linear model (GLM) analysis. A random-effects analysis within the framework of the GLM was applied to model event-related responses (Friston et al., 1995).

Four regressors were defined for each participant based on the onsets of the relevant trials: “*Conformity to In-group*,” “*Conformity to Out-group*,” “*Non-Conformity to In-group*,” and “*Non Conformity to Out-group.*” Brain responses were time-locked to the presentation of the estimate of either the in-group or out-group member. Regressors were modeled with a canonical hemodynamic response function and a GLM analysis was used to create contrast images summarizing differences in brain activity across the *Conformity to In-group* and *Non-Conformity to In-group* trials, as well as differences in brain activity across the *Conformity to Out-group* and *Non-Conformity to Out-group* trials. To test hypotheses regarding brain areas that were uniquely involved in conformity to an in-group member, we masked the brain activity present in the In-group contrast map (*Conformity to In-group* > *Non-Conformity to In-group*) with the Out-group contrast map (*Conformity to Out-group* > *Non-Conformity to Out-group*) (*p* < 0.05 uncorrected) (e.g., Pochon et al., 2002; Uncapher et al., 2006; Enzi et al., 2009). This exclusive masking procedure revealed activity in the In-group contrast map that did not overlap with the brain areas involved in the Out-group contrast map (*p* < 0.001, uncorrected, 10-voxel minimum). To assess whether there was a relationship between brain activity underlying conformity and the self-report measures assessed, we correlated individual beta values of the reported brain activity with participants' scores on the scales measuring identification, positive associations, and trust toward in-group and out-group members.

## Results

### Manipulation check

In line with the group manipulation, participants identified more strongly with in-group members (*M* = 4.7, *SD* = 1.0) than with out-group members (*M* = 3.2, *SD* = 1.0), *t*_(23)_ = 5.4, *p* < 0.001 (paired *t*-test). There were no differences in identification between the two out-groups, that is, participants identified equally with out-group members that were classified as background perceivers (*M* = 3.3, *SD* = 1.1) or that were not classified (*M* = 3.2, *SD* = 1.2), *t*_(23)_ = 0.6, *ns*. Consistent with an in-group preference, participants had more positive associations with in-group members (*M* = 5.8, *SD* = 0.6) than with out-group members (*M* = 4.9, *SD* = 0.9), *t*_(23)_ = 4.3, *p* < 0.001, and participants reported greater trust in in-group members (*M* = 4.9, *SD* = 1.1) than in out-group members (*M* = 4.1, *SD* = 1.0), *t*_(23)_ = 3.3, *p* < 0.005.

### Behavioral conformity

Participants conformed more often to in-group judgments than to out-group judgments. The percentage of trials in which participants' judgment matched the estimate of the group member was higher when seeing the estimate of an in-group member (*M* = 67.8%, *SD* = 9.4%) than an out-group member (*M* = 65.4%, *SD* = 9.2%), *t*_(23)_ = 2.8, *p* < 0.01. In-group conformity did not differ between easy (≤11 dots), and difficult trials, *t*_(23)_ = 0.5, *n.s*.

### Neural correlates of in-group conformity (Table 1)

**Figure 2:**
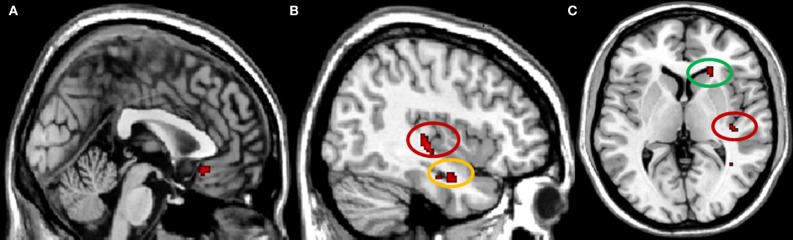
**Brain regions involved in in-group conformity, *p* < 0.001 uncorrected. (A)** subgenual ACC, *x* = 0; **(B)** pSTS/insula (circled in red) and hippocampus (circled in yellow), *x* = 40; **(C)** pSTS/insula (circled in red) and caudate (circled in green), *z* = 4.

When examining brain areas exclusively involved in conformity to the in-group, we found a significant increase in activity in right caudate, subgenual anterior cingulate cortex (subACC), right hippocampus, and in the intersection of the right posterior insula and the posterior superior temporal sulcus (pSTS) (Figure 2). Analyses of the In-group contrast (*Conformity to In-group* > *Non-Conformity to In-group*) and Out-group contrast (*Conformity to Out-group* > *Non-Conformity to Out-group*) directly did not reveal any significant activation patterns. Next, we calculated whether there were any correlations between the neural activity underlying in-group conformity and participants' self-reports on in-group trust and associations. Correlation analyses were conducted for each brain region found to be involved in in-group conformity, and corrected for multiple comparisons accordingly (Bonferroni-corrected *p* < 0.0125). We found that the activity in the posterior insula/pSTS positively correlated with participants' scores on the trustworthiness of in-group members (*r* = 0.53, *p* < 0.01). Thus, the more trustworthy participants' judged their in-group, the higher the activity in this region. No other significant correlations were found.

**Table 1:** **Significant areas of activation associated with conformity toward in-group members**.

**Brain region**	**HEM**	**BA**	***x***	***y***	***z***	**Nr of voxels**	**Max *Z*-score**
Hippocampus	R	20	36	−10	−18	101	4.6
pSTS/insula	R	48	40	−20	0	68	4.6
SubACC	-	11	0	26	−8	28	3.7
Caudate	R	47	22	29	4	21	3.6

MNI coordinates of peak activity. HEM, hemisphere; BA, Brodmann area; pSTS, posterior superior temporal sulcus; SubACC, subgenual anterior cingulate cortex.

## Discussion

To examine the basic processes that mediate in-group conformity, we explored the neural mechanisms underlying this effect. Activity in the caudate was selectively enhanced when participants conformed to the in-group, supporting the hypothesis that the striatum plays an important role in social influence (Klucharev et al., 2009; Campbell-Meiklejohn et al., 2010; Zaki et al., 2011). The striatum, located in the center of the brain, is a major input station for midbrain dopamine neurons and plays a primary role in the processing of rewards, including primary rewards such as liquids, foods, and sexual stimuli (Redouté et al., 2000; Berns et al., 2001; O'Doherty et al., 2003), as well as to money (Knutson et al., 2000) and more abstract rewards such as reputation or status (Izuma et al., 2008; Zink et al., 2008). The finding that the striatum is involved in in-group conformity, in conjunction with conformity-related activations in other low-level processing areas such as the subACC, an area implicated in the experience of affective states (Drevets et al., 1997), and the hippocampus, an area important for the retrieval of spatial memories (such as the dot display) (e.g., Eldridge et al., 2000), suggests that in-group conformity is mediated by fundamental value signals in the brain. Importantly, involvement of the subACC suggests that affective signals may be related to the positive experience of social inclusion in particular, as this brain region has been implicated in social acceptance (Somerville et al., 2006), and also shown to be more active for individuals low in rejection sensitivity (Burklund et al., 2007). Taken together, these findings suggest that people conform more to in-group members than to out-group members because the behavior of in-group members is more strongly associated with the experience of positive affect and reward.

Greater activity for in-group conformity was also found in a region bordering the pSTS and the posterior insula, with peak activity in the posterior insula but extending further into pSTS. The pSTS is an area often implicated in the cognitive capacity of perspective taking, typically termed Theory of Mind (Frith and Frith, 2006). The concept of Theory of Mind is defined as the understanding that others have their own individual perspective on the world, which may differ from your own. Finding that the BOLD response in the pSTS is selectively enhanced for in-group conformity is interesting, as this could imply that participants took the perspective of the other more when the other was an in-group member than an out-group member. This hypothesis supports previous work suggesting that people mentalize more about in-group than out-group members (Harris and Fiske, 2006; Freeman et al., 2010; Heatherton, 2011). Moreover, activity in the pSTS correlated with participants' self-report measures on the perceived trustworthiness of the in-group, again indicating that those who reported strong feelings of trust toward their in-group were more in-tune with the mental state of their in-group member.

The present findings complement behavioral studies (e.g., Asch, 1951; Deutsch and Gerard, 1955; Cialdini and Goldstein, 2004; Jetten et al., 2004) and recent pharmacological work (Stallen et al., 2012) on group influence, and expand on investigations of the neural bases of both conformity (Berns et al., 2001, 2010; Klucharev et al., 2009, 2011; Mason et al., 2009; Campbell-Meiklejohn et al., 2010; Zaki et al., 2011; Berns and Moore, 2012), and in-group influence (Volz et al., 2009; Hein et al., 2010). Furthermore, our results provide potential relevant insights for the design of social influence programs. We show that conformity to the in-group is presumably mediated by both positive affect as well as perspective taking. This suggests that social influence programs may benefit by emphasizing the positive aspects associated with in-group membership rather than, for instance, stressing the negative feelings associated with social exclusion. Additionally, our data suggest that social influence-programs will work more effectively when the target is stimulated to imagine the state of mind of the in-group and “puts himself in the others” shoes', thereby facilitating perspective-taking processes which may result in more trust directed toward in-group information.

Future research could productively test these hypotheses, as the present effects are small and the interpretations here are based on previous research linking activity in specific brain regions to basic cognitive functioning. In general, the ability to assess with certainty the cognitive processing reflected by specific brain activity is challenging due to the multiple functions brain regions typically engage in (Poldrack, 2006). Follow-up behavioral and neuroimaging studies can reveal how the basic mechanisms of in-group conformity reported here are modulated by different contexts, in particular participant population and decision-making domain. For instance, the conformity effect reported here is quite small, likely due to the minimal conditions under which in-group conformity was tested. However, when using natural groups, such as friends or sports teams, and when measuring conformity in a decision-making domain more closely related to identity formation, such as consumption choice for clothing, music, hairstyle, or food (Bearden and Etzel, 1982; Berger and Heath, 2007, 2008), the present in-group conformity bias would likely be stronger. In-group conformity in contexts more relevant to identity formation may not only be mediated by mechanisms of positive affect and perspective taking as reported here, but by the activation of social identity processes as well. A candidate brain region for these processes is the dorsal medial prefrontal cortex, as previous research has found this area to be implicated in the activation of self and group identity and to correlate with a behavioral in-group bias (Volz et al., 2009). In addition, our findings encourage the study of in-group conformity across different age ranges. We found in-group conformity to be mediated by increased activity in the subACC, an area known to be involved in social inclusion (Somerville et al., 2006; Burklund et al., 2007) and positive affect (Kim et al., 2003; Sharot et al., 2007) in adults. However, in adolescents the subACC has been found to correlate with social exclusion (Masten et al., 2009). This may predict that while in-group conformity in adults is primarily driven by the positive affect associated with social inclusion, in-group conformity in adolescents might be driven more by the negative affect associated with social exclusion. Social influence campaigns targeted at adolescents may therefore be more effective when emphasizing the negative aspects of social exclusion than the positive affect associated with social inclusion.

## Conclusion

The present findings complement recent work on the physiological bases of both conformity (Breiter et al., 2001; Klucharev et al., 2009, 2011; Mason et al., 2009; Berns et al., 2010; Campbell-Meiklejohn et al., 2010; Zaki et al., 2011), and in-group influence (Volz et al., 2009; Hein et al., 2010; Stallen et al., 2012). The current study is a first step toward understanding the nature of actual in-group conformity behavior, and provides a first insight into what mechanisms may drive this effect. Our data indicate that both positive associations linked to in-group members, as well as the ability to take the perspective of the in-group, likely play an important role in in-group conformity. Understanding why group membership has such a profound influence on decision-making provides a window into one of the basic motivations underlying people's behavior, and may help in developing effective campaigns based on a social influence approach.

### Conflict of interest statement

The authors declare that the research was conducted in the absence of any commercial or financial relationships that could be construed as a potential conflict of interest.
